# Ethical issues in human genomics research in developing countries

**DOI:** 10.1186/1472-6939-12-5

**Published:** 2011-03-18

**Authors:** Jantina de Vries, Susan J Bull, Ogobara Doumbo, Muntaser Ibrahim, Odile Mercereau-Puijalon, Dominic Kwiatkowski, Michael Parker

**Affiliations:** 1 The Ethox Centre, Department of Public Health and Primary Care, University of Oxford, Old Road Campus, Headington, Oxford, OX3 7LF, UK; 2 The Wellcome Trust Centre for Human Genetics, University of Oxford, Roosevelt Drive, Oxford, OX3 7BN, UK; 3 Malaria Research and Training Centre, Faculty of Medicine, Pharmacy and Odonto-Stomatology, University of Bamako, PO Box: 1805 Point G, Bamako, Mali; 4 Institute for Endemic Diseases, University of Khartoum, Medical Campus, Qasser Street, PO Box 102 Sudan; 5 Institut Pasteur, Unité d'Immunologie Moléculaire des Parasites, 28 Rue du Dr Roux, 75724 Paris, Cedex 15, France; 6 Wellcome Trust Sanger Institute, Hinxton, Cambridge, CB10 1SA, UK

## Abstract

**Background:**

Genome-wide association studies (GWAS) provide a powerful means of identifying genetic variants that play a role in common diseases. Such studies present important ethical challenges. An increasing number of GWAS is taking place in lower income countries and there is a pressing need to identify the particular ethical challenges arising in such contexts. In this paper, we draw upon the experiences of the MalariaGEN Consortium to identify specific ethical issues raised by such research in Africa, Asia and Oceania.

**Discussion:**

We explore ethical issues in three key areas: protecting the interests of research participants, regulation of international collaborative genomics research and protecting the interests of scientists in low income countries. With regard to participants, important challenges are raised about community consultation and consent. Genomics research raises ethical and governance issues about sample export and ownership, about the use of archived samples and about the complexity of reviewing such large international projects. In the context of protecting the interests of researchers in low income countries, we discuss aspects of data sharing and capacity building that need to be considered for sustainable and mutually beneficial collaborations.

**Summary:**

Many ethical issues are raised when genomics research is conducted on populations that are characterised by lower average income and literacy levels, such as the populations included in MalariaGEN. It is important that such issues are appropriately addressed in such research. Our experience suggests that the ethical issues in genomics research can best be identified, analysed and addressed where ethics is embedded in the design and implementation of such research projects.

## Background

Recent years have seen an explosion of scientific interest in the use of human genomic variation to study common complex diseases. The hypothesis is that human genetic diversity can be used as a tool to study the causal mechanisms of disease. Examples include Genome-Wide Association studies (GWAS) and, more recently, projects that make use of next-generation sequencing. Over the past 5 years, GWAS have proven very valuable in identifying regions of the genome that affect resistance or susceptibility to a wide range of common diseases, although the method provides simply a starting point, and a range of other approaches will be required in future to fully characterise and understand the complex genetic determinants of human health and disease. To date, whilst many such studies have taken place focussing on a wide range of conditions, hardly any of these have been applied to diseases that primarily affect people in lower income countries[1,2].

There are good ethical reasons for encouraging medical research on diseases affecting populations with lower average income and literacy levels. Substantial global inequalities exist in health measures such as mortality, quality of life and disease incidence. These persist despite increasing levels of overall wealth [3,4]. Even today, only a small proportion of medical research focuses on the problems primarily affecting the world's poorest people [5]. Applying the methods of genomics research to these diseases is one way to address this imbalance.

Genomics research raises a number of ethical challenges wherever it is carried out [6,7]. Some of the issues identified in the literature to date include consent [8,9], privacy [10-12] and the collection, storage and release of genomic data [13,14]. Despite the existence of a substantial and developing literature on the ethical issues arising in genomic studies, this literature has as yet not adequately addressed the specific ethical challenges presented by genomics research which takes place in the context of collaborative global health partnerships between high and low income countries. This paper aims to begin to address this gap by reporting on the ethical challenges that have been identified in the development of the Malaria Genomic Epidemiology Network (MalariaGEN), a partnership of malaria researchers in over 20 countries supported by the Grand Challenges in Global Health Initiative [15].

Malaria is a so-called complex disease, involving an intricate immunological pathway and dynamic relationships between the human, the mosquito and the malaria parasite [16-18]. Malaria is one of the leading causes of infant mortality in tropical countries, causing the deaths of nearly 1 million children under five in 2006 [19], as well as debilitating illness in a quarter of a billion people worldwide [19,20]. By combining large-scale epidemiological studies with state-of-the-art genomic technology, GWA studies hope to use human genetic diversity as a tool to study the causal mechanisms of disease [17]. MalariaGEN research is conducted in several countries in Africa, Asia and Oceania. An overview of study sites can be found on the project website, http://www.malariagen.net. Whereas our experience is relevant to populations in these countries, we believe that what essentially distinguishes the MalariaGEN research from other genomic research conducted in wealthier parts of the world, is lower average income and literacy levels. Our findings may therefore be equally relevant to genomic research conducted on poorer populations in other parts of the world.

## Discussion: Ethical Issues in Human Genomics Research in Lower Income Countries

### i. Protecting the interests of research participants

Whilst genomics research presents important ethical challenges in the recruitment of participants regardless of where it is conducted, prospective participants in lower income countries are more likely to be poor and to have limited access to healthcare, education and other resources. This means that the carrying out of research in these setting invariably presents challenges of a different order to those in higher income countries. In this section, we explore the particular challenges presented by genomic research for community participation and the obtaining of valid consent.

#### Community engagement

Driven both by recognition of the need for locally relevant health research in lower income countries, and by awareness of the potential for exploitation in contexts of vulnerability and inequality [21], collaborative partnership and social value have been proposed as benchmarks against which the ethics of research in lower income countries should be measured [22,23]. The empirical literature that exists on community partnership tends to show that the achievement of these benchmarks may only really be possible in the context of effective and sustained community engagement and accountability [24-26]. Community engagement strategies therefore are increasingly seen as a key element of ethical best practice in research. Despite this growing emphasis on the importance of community engagement however, there is still relatively little published experience or evaluation of engagement in practice. There is certainly a need for more research in this area, and for the sharing of models of good practice.

Notwithstanding its importance, the actual achievement of successful and appropriate community engagement presents a number of practical and ethical challenges. Some of these relate to the question of how the relevant community is to be identified and represented [24,27]. Others concern the identification and establishment of procedures, principles and mechanisms of engagement that are fair, inclusive, accountable and appropriate to the research setting [28,29].

In the course of its development, a number of issues specific to international collaborations were identified by the MalariaGEN Network. One of these concerned the relationship between the need to establish shared good community engagement practices across the network on the one hand, and the need for sensitivity to local variation on the other. MalariaGEN studies were conducted across a wide range of settings, spanning from referral hospitals in urban areas to traditional rural villages. Each of these settings involved very different kinds of 'communities', all with their own decision-making strategies.

In the context of such diversity, identifying common ground where researchers can share experiences and insights is inevitably challenging. The extent to which 'best practice' can be shared and translated across such contexts will depend on a wide range of factors particular to the nature of the research and its location(s). In practice, MalariaGEN researchers across the network engaged in some form of community engagement, but the majority of such initiatives were developed ad hoc by researchers in the absence of agreed-upon guidelines for best practice or even a toolkit for community engagement. The limited guidance currently available presents important challenges for researchers in meaningfully engaging with research communities.

Another practical challenge in the context of community participation in genomics research concerns how to explain to communities what the study involves in ways that are accessible and make understanding and engagement possible. Some suggestions have been made about how to explain key terms of genomic studies [30], but the extent to which such abstractions effectively explain the risks and benefits involved remains yet to be seen. Particularly challenging is the fact that many of the potential harms and benefits of genomics research relate to populations rather than to individuals [31]. Whether this is an important feature of genomic studies for communities and participants, and how it could best be explained and discussed, requires further investigation.

Within the context of MalariaGEN, we did not resolve ways of addressing these challenges. The complexity of community engagement exercises is widely acknowledged [24,27,32], and there may not be a one-size-fits-all solution. But in our experience, these are important challenges in the context of international collaborative genomics research, and are going to require careful attention in the establishment and maintenance of successful research networks. Scientific researchers cannot be expected to face these challenges alone, and constructive collaboration is needed with others knowledgeable in local community structures and processes and ethics, preferably as an integral part of the research process.

#### Valid consent

Valid consent for research participation must be adequately informed and understood, voluntary, and given by someone who is competent to do so [33]. It also requires the processes through which consent is obtained to be locally appropriate [34]. Designing and implementing consent processes in the context of collaborative genomics research on populations characterised by lower average income and literacy levels presents many challenges. Some of the difficulties relating to the development and implementation of consent processes in lower income countries have been discussed in the literature [35-38]. However, little of this addresses the particular issues presented by the novel field of genomics research.

A critical challenge to achieving valid consent in lower income countries arises in relation to providing appropriate information to participants in a comprehensible manner [39]. In genomics, challenges are presented by the need to explain concepts such as 'genetics', 'genomics', 'data release', and the reasons underlying the need to collect large numbers of healthy population controls [28]. It may be possible to link these concepts to local knowledge of genetics, for instance that particular facial traits are often inherited within families [40]. But although it may be possible to explain some aspects of genomics research in ways that are not entirely alien to research participants, ensuring that participants give valid consent for genomic studies remains a significant challenge.

There are other challenges to obtaining valid consent for genomics research. Information produced by genomic research has, for example, the potential to be informative about people other than the research participant. There has been much discussion about the importance of privacy protection for individual research participants in genomic studies [41-43]. Where personal identifiers are removed from genomic datasets there may arguably be limited risk of participant identification. Yet even where this is the case, there remains a possibility that unwanted information about populations, communities or families will be revealed. At the population level, GWA studies have for example the potential to reveal that a stigmatising condition is more likely to occur in one population than another [8]. In that sense, it raises the possibility that genomic data could be used in ways that would have adverse effects for the populations involved, possibly through generating research results that could be used to stigmatise groups based on their genetic make-up [44,45]. There may be a need to take this kind of issue seriously when designing consent processes for genomics.

The MalariaGEN Network has sought to address some of these challenges through the collaborative development of an informed consent template and guidelines for informed consent http://www.malariagen.net/home/ethics/consentpolicies.php. These were developed by and for MalariaGEN researchers and in consultation with some ethics committee members. In addition, the MalariaGEN ethics team (JdV, SB and MP) discussed the consent procedures, challenges and best practice with researchers and fieldworkers at several research sites. Particular challenges with regard to consent were how to collect consent in emergency situations, and how to explain the rationale for collecting samples from healthy children. A key strategy of genomic studies, like the MalariaGEN study, is comparing genomic data between children that are ill with malaria (the cases) and healthy children (the controls). The collection of both types of samples raised issues with regard to consent. Namely, children presenting at the hospital were often severely ill and required immediate medical assistance. Timing of consent in this emergency setting presented a real challenge in these cases. On the other hand, the genomic study requires the collection of blood samples from healthy children as well. Explaining the reason for collecting blood samples from healthy children also constituted important challenges for the MalariaGEN study. The complexities of resolving these issues were such that further empirical research was conducted at two MalariaGEN research sites, with the aim of investigating how best to obtain consent for genomic studies in low income countries. Papers reporting on the results of these studies are forthcoming.

### ii. Regulating human genomics research

Where genomics research focuses on diseases affecting populations with lower average income and literacy levels, it tends to take place in collaborations between researchers from higher and lower income countries. For example, whereas the infrastructure for genotyping and whole genome analysis is usually based in higher income countries, the patients affected by the diseases are based in lower income countries. This distribution of research resources raises important issues about the use of archived samples, sample ownership and ethics review by multiple committees.

#### Sample export and ownership

Because GWA studies require access to sophisticated laboratories and large-scale genotyping facilities with attendant statistical expertise, most of which are currently only available in a few countries in the world, samples collected for GWA studies are often exported for processing, quality control and genotyping. The export of samples from lower income countries is increasingly seen to present important ethical issues both by researchers and by research ethics committees [46]. Many of these arise from concerns from researchers and ethics committees in the samples' country of origin that once samples have been exported, their control over subsequent use will be limited [47,48]. At least part of the concern is that whilst samples were often collected for a particular purpose on the basis of a relationship of trust between researcher and research participant, this relationship of trust may become diluted when samples from different studies are used in research abroad by scientists who have never met the participants nor are familiar with the samples' country of origin. Upshur and colleagues argue that important contextual understandings such as the cultural value of tissues and samples may get lost if researchers who have no knowledge of the culture prevalent in the place in which samples were collected, perform the majority of work and analysis on the samples [47]. The absence of agreed-upon policies for export, sample handling and destruction [48,49], as well as the fear that samples may take on monetary value in international research [50], can aggravate such concerns.

A key challenge for genomics research conducted on populations with lower average income and literacy levels is how conditions can be created under which it is possible for researchers and research ethics committees to feel confident that samples and data will be used appropriately, and that the decision-making process for their use is sufficiently transparent. In MalariaGEN, the export of samples was found to be a particular stumbling block for ethics committees. The committees required detailed information on the need to export samples, as well as descriptions of the exact sample handling. Specific Material Transfer Agreements (MTAs, Case 1) describing in detail the nature of the work to be carried out in foreign laboratories, as well as procedures for sample return or destruction at the end of the project, were instrumental in addressing some of these concerns. In some instances, submission of signed MTAs was a requirement for ethics approval.

## | Case 1 | Material Transfer Agreements

A Material Transfer Agreement is a contract between two parties involved in a research project that specifies exactly the nature of work that is to be done on materials given by the one party to the other. It typically consists of specifications of the following elements:

• *the materials to be transferred;*

• *the exact work to be done on the materials;*

• *the conditions of storage of the materials, including for instance details on building access and security;*

• *the people that are to work with the samples, typically the heads of research groups and all the members of their group;*

• *the duration of the collaboration;*

• *an agreement about data sharing and collaboration in analysis;*

• *procedures for agreeing on any other work that is not covered in the current MTA*.

*MTAs are an important means of protecting the interests of the researchers collecting and supplying samples. In MalariaGEN, they were instrumental in addressing the concerns of ethics committees regarding ownership, long-term storage and sample re-use*.

It is important to remember, however, that whereas MTAs can play a significant role in organising the legal responsibilities in the research process, they cannot accommodate all the above challenges. In particular, issues of perceived symbolic (non-legal) ownership over DNA, and concerns about whether a populations' genetic material relates to its identity, have long played an important role in genetic and genomics research [51-53]. In addition, where genomics research takes place in the context of resource inequalities between research partners, the concentration of samples and resources in particular partner sites raises questions about fairness, ethical oversight, benefit sharing, and long-term development of capacity at all research sites.

In MalariaGEN, a number of attempts have been made to address these more exacting challenges, in addition to MTAs and research contracts. First, the network developed a capacity building scheme in which young researchers from all partner sites were trained in the analysis of genomic data (see section Capacity Building). Second, the network recognised the need to enable all contributing researchers to analyse their own data before it was made publicly available and incorporated this into the MalariaGEN Data Release Policy http://www.malariagen.net/home/downloads/16.pdf. Third, the network sought to develop software that allows the remote analysis of genomic data - meaning that MalariaGEN researchers anywhere in the world could analyse data without the need to invest in expensive in-house infrastructure for data analysis and storage. Funding has also been obtained to fund a PhD studentship to explore the issues relating to the collection, storage and export of biological samples and to work towards the development of recommendations on good practice.

### Archived samples

Successful GWA studies require the collection of very large numbers of samples and well-characterised phenotypes. This is labour-intensive and time consuming, even in well-resourced health systems. Moreover, the conditions and lack of infrastructure for treatment and research in lower income countries are often not conducive to the rapid collection of the very large numbers of samples required for genomic studies. Significant improvements in malaria treatment and prevention [54,55] also mean that fewer samples are available for research on that disease. Taken together, these factors mean that GWA studies such as MalariaGEN need to rely heavily on the use of archived samples.

The use of archived samples raises a number of important ethical challenges. The relatively recent development of genomics means that it is unlikely that consent to studies conducted a few years earlier would have included it. Questions therefore arise about how decisions about the re-use of such samples should be made and who should be making them. One issue concerns the legitimacy of ethics committees to represent the views of research participants and to decide on re-use on their behalf [48,49,56]. Further issues arise about whether there are cases in which re-consent should be required or whether there are forms of research for which new samples should be collected altogether. A balance may need to be struck between the ethical implications of collecting many thousands of new samples against the ethics of using archived samples with less than ideal consent.

For MalariaGEN, almost all contributed samples were originally collected for research on malaria, but only a subset was collected with consent for genomic or genetic studies. All ethics committees reviewing the MalariaGEN studies approved the inclusion of archived samples in the prospective genomic study. Only in one site did researchers decide to re-consent participants whose samples were collected many years ago, but this was mainly because the research design of the particular sub-study required taking additional samples.

### Ethics review

International collaborative research projects need to obtain ethics approval from a multitude of committees from around the world. This can be a daunting task, especially where committees express different or conflicting points of view or place additional requirements on researchers [57], such as changes to a consent form that has already been approved by another ethics committee.

The review of genomic studies is challenging: the science is difficult to comprehend [58], the studies are hypothesis-generating rather than hypothesis-testing, use very large sample numbers [59,60], and generate very large amounts of data that can be analysed many times for different purposes. In addition, genomic studies take place in a context where data sharing is the norm [61]. This raises ethical challenges that may not be familiar to members of ethics committees. Ethics committees reviewing research in this area may not have had the training or experience to enable them to identify and analyse the key ethical issues - a well-recognised problem also in other research fields [62-64]. In addition, many of the key ethical challenges of GWA studies have only been identified fairly recently. Pertinent issues like consent and privacy remain the topic of debate [43], and consensus about the best approach to accommodate these challenges in research has not been reached.

It is therefore hardly surprising that obtaining ethics approval for the various MalariaGEN studies was a significant challenge. Over twenty ethics committees in sixteen countries reviewed and approved the study, with review taking up to a year to complete at some partner sites (see Case 2). For many of these committees, the MalariaGEN study was one of the first genomic studies to be reviewed.

The MalariaGEN study was approved by all ethics committees, although in some cases the approval process consisted of multiple rounds of correspondence to clarify study design and rationale. Overall, there was considerable homogeneity in the concerns raised by committees in various countries. The main points raised concerned: how to ensure that participants give valid (informed) consent; justifications for the export of samples and specification of the procedures for sample return or destruction at the end of the project; ensuring the appropriate recognition of local investigators' contributions and capacity development; ensuring that genomic data will not be used to harm populations or countries; and ways of assigning benefits to the country or community that donated the samples.

A particular challenge relates to fast increases in the number of genetic variants that can be reasonably genotyped for a project like MalariaGEN. Genomics is a fast-moving field and new technological opportunities are developed monthly. These ought to be exploited to maximise the power of genomic studies; yet they may mean that the ethics approval is outdated.

MalariaGEN adopted two ways of approaching the challenge of obtaining ethics review. First, it held a number of ethics workshops to which members of some of the ethics committees were also invited. In this way, MalariaGEN received some very important feedback about what were perceived to be the key ethical challenges by ethics committee members, which could in turn be integrated into project policies and proposals. Second, when the Network was seeking to address particular issues, such as data sharing, it sought to establish working relationships with ethics committees to receive feedback on proposed policies. This enriched the Network's thinking about particular ethical challenges relating to the MalariaGEN studies.

## | Case 2 | Ethics Review and the Malariagen Ethics Team

*The MalariaGEN Network has adopted an integrative approach to ethics, with a team of ethicists working alongside researchers to identify and address ethical challenges relevant to the scientific project. One way in which this approach has benefitted the Network is in providing assistance during ethics review. In consultation with project members, the MalariaGEN ethics team developed template information about the project, including information about the methodology, preliminary data sharing principles and ethical issues. This information was subsequently used by many local researchers in their MalariaGEN ethics application*.

*The MalariaGEN ethics team also provided assistance in interpreting and answering any queries that ethics committees raised in the review process, or in liaising between project management and ethics committees in addressing local concerns. Lastly, the ethics team provided a bridge between ethics committees and researchers in addressing pertinent ethical challenges at later stages in the project. Most importantly, it facilitated a discussion of ethical considerations important in the context of data sharing*.

### iii. Protecting the interests of scientists in the developing world

A fundamental principle underlying many genomics research projects is that data should be shared with third party researchers for secondary analysis. Whereas this principle may be ethically laudable in promoting the utility of data, it is also based on an assumption that all researchers have equal access to the data, and comparable facilities and abilities to analyse it. This assumption does not hold for all researchers, and especially not for those based in low-income countries. A specific focus on equality and fairness may be necessary to protect the interests of those researchers.

#### Data sharing and data release

There are strong scientific and ethical arguments for sharing genomic data as the full scientific value of a GWA study may not be realised unless it is analysed by different methods and combined with other datasets. Despite the ethical importance of promoting the availability of genomic data to the scientific community [65], moves towards open access have also generated a significant literature concerning the compatibility of open access with important ethical principles and values [61]. The range of ethical issues identified is extensive. It includes concerns about: privacy and whether anonymity can be guaranteed [10]; data security [11]; the implications of collecting and storing vast amounts of data and its uncertain future use; the implications of data release for populations and for family members of participants [7]; the need to strike a proper balance between research and protection [65]; the development of appropriate governance mechanisms [31]; the implications for trust, consent and autonomy [45,66]; commercialisation; and the ethical importance of the sustainability of databases.

While MalariaGEN was founded with open access in mind [13] it was clear that the development of an effective, appropriate approach to GWA data release required widespread consultation across the network and with external stakeholders. After extensive discussion within the Network, and consultation with parties outside it, it was concluded that it would be inappropriate to provide unrestricted public access to GWA data on individuals accompanied by specific phenotypic data. Following consultation, it was agreed that access to MalariaGEN datasets would be mediated via an independent data-access committee and that researchers would be granted access to genotyping data and to a limited amount of clinical and demographic data only after signing a legally-binding data-access agreement which placed restrictions on the acceptable uses to which MalariaGEN data can be put [14]. The principles set out in the MalariaGEN Data Access Policy incorporate key ethical principles that MalariaGEN researchers agreed should be respected by any third party using the genomic data. Particularly important factors in deciding to regulate data access were the potential for genomic data associated with ethnicity to lead to ethnic stigmatisation and the importance of ensuring that future data uses are compliant with the purposes for which the samples were collected.

Another key reason for adopting a managed approach to data release was the view that an ethical data release policy must also be combined with adequate protections for researchers in lower income countries. Where data is open access, training and capacity building activities are necessary to ensure that researchers in the lower income countries have a fair chance to publish their analyses of the study [14].

#### Capacity building

A significant challenge for sustainable GWA studies in lower income countries concerns the development of research capacity across participating research sites. Where researchers are engaged in the collection of large numbers of samples, it is vital that they are also in a position to analyse research results, and to use their contribution for career development. Capacity building would also allow scientists to enlarge their analyses to additional, locally-held phenotypes or to build on key genomic findings to develop further research projects. Such projects could aim at understanding the biological mechanisms relating to the genetic effect, or undertaking additional genetic studies. For GWA studies, capacity building is also imperative as benefit sharing as the main outcomes of the research are likely to be expertise and knowledge, rather than treatments.

After genotyping or sequencing is completed, GWA studies are strongly reliant on bioinformatics and computational technology and infrastructure, but not all these need to be present at the site where analysis is conducted. Especially when data is accessed from a distance, genomic analysis is relatively affordable. Thus, genomic methodology may offer excellent opportunities for real and sustainable involvement from researchers in lower income countries (See Case 3). A key bottleneck, however, is the ability to work with genomic data.

## | Case 3 | Capacity Building in Genomics in Developing Countries

The relative affordability of genomic analyses, once data has been generated, may mean that this research methodology lends itself well for successful capacity building. A key aim of sustainable GWAS ought to be that all researchers are able to analyse and publish analyses of their data. However, in order to ensure that all research sites are capable of conducting site-specific analyses, the following need to be considered:

**Central Data Repository: ***Genomic data files are large and require very significant computational resources for confidential storage. For successful local analyses, it may not be necessary to store the data at all research sites; rather, a central data repository may be a solution. A prerequisite, however, is that such a repository can easily and securely be accessed at a distance, and that tools exist that allow for relevant data to be extracted and/or analysed when necessary. It also requires a trusted body to maintain and share the data*.

**Local Infrastructure: ***Ideally, investigators around the world should be in a position to analyse genomic data, and combine it where appropriate with clinical data held locally. This would maximise the utility of genomic data, provide career incentives, and possibly generate new research findings that are specific for the populations that donated samples. But such analyses will only take place if sufficient resources are available locally to support them. Necessary for local analyses are*

• *effective high-speed connectivity to the Internet;*

• *high speed computers;*

• *human resources including well-trained IT staff and bioinformatics scientists;*

• *a supportive institutional environment that allows staff to acquire the necessary skills, and that provides career opportunities*.

**Network Infrastructure: ***Where research takes place in international collaboration, then the network should identify strengths and weaknesses for all partner sites, and be committed to sharing these with others. In particular, the network should seek to support local analyses, for instance by making a data analyst available to all sites. Also, regular meeting opportunities to discuss particular challenges, as well as a mentoring scheme to support junior researchers may be considered*.

In the context of MalariaGEN, this challenge was addressed in a training programme in which junior researchers from the participating research centres received intensive training in the analysis of genomic data [15]. They participated in several data analysis workshops and were also invited to the annual MalariaGEN meetings where they presented site-specific analyses. In addition, they received support to develop and submit conference abstracts, and a sub-group also received support to apply for PhD fellowships.

While this capacity building programme was successful in engaging young local researchers in genomic studies, this is also a vast and difficult area that requires long-term investment and commitment from all parties in research. On the contrary, research projects often have to rely on short-term research funding and have milestones that need to be met. Also, it can only be expected to be successful if linked into a supportive and stable institutional environment. To train a group of enthusiastic and young people in the scope of a few years to a standard where they can successfully analyse genomic data is then a significant challenge.

Second, although software can be developed which allows researchers to conduct analyses at a distance, it inevitably requires stable Internet connections. Although the majority of research centres around the world now have fairly stable access to the Internet, some centres remain unconnected. In the context of an international collaborative research project it is unfeasible to develop computational and networking capacity at all the research sites, which means that some sites may remain at a disadvantage in terms of data utilization and analysis. Site visits and local exchange of facilities may be a solution to this problem.

## Summary

The application of GWA methodology to poverty-related diseases presents a wide range of ethical challenges. As our experiences with MalariaGEN have shown, the three key challenges highlighted in the literature to date - namely consent, privacy and data release - are by no means an exhaustive list. The ethical issues relevant to GWA studies conducted on populations with lower average income and literacy levels also include issues around the inclusion and reuse of archived samples, export of samples, ethical review and capacity building. We believe that identifying and addressing ethical considerations should be integral to the development of responsible whole-genome studies in recognition of the need to develop appropriate responses to issues that are relevant to populations in low-income countries.

The fact that collaborative genomics research in lower income countries involves the establishment of large and diverse scientific networks bringing together diverse and interdependent forms of expertise and institutions in higher and lower income countries, means that responsibility for the ethical dimensions of such research is inevitably shared. Important issues such as ownership of samples and data and capacity to analyse genomic data need to be addressed for such studies to be successful. In addition to establishing means of developing consensus about ethical issues to be addressed in their research, networks need to determine how best to tailor the implementation of ethical principles to individual research sites.

In this paper, we have described the experiences of exploring and addressing the ethical issues that emerged in the context of the research of the MalariaGEN Network. But one size does not fit all and the development of appropriate responses to these ethical issues will need to be conducted on a case by case basis. What will link international genomics research projects, though, are the more general principles of justice, ownership and the fair distribution of resources. Given the urgent need to alleviate the disease burden for people living in lower income countries, and the tremendous opportunity to redress wider issues of global injustice and inequality, the need to tackle and resolve the ethical challenges surrounding GWA studies is as important as it is acute.

## Abbreviations Used

GWA: Genome Wide Association; MalariaGEN: Malaria Genomic Epidemiology Network; MTA: Material Transfer Agreement

## Competing interests

The authors declare that they have no competing interests.

## Authors' contributions

JdV, SJB and MP prepared the manuscript. OD, MI, OMP and DK commented on all versions and made substantial contributions to the final draft of the paper. All authors read and approved the manuscript before submission.

## Pre-publication history

The pre-publication history for this paper can be accessed here:

http://www.biomedcentral.com/1472-6939/12/5/prepub
